# On the steps of cell-to-cell HIV transmission between CD4 T cells

**DOI:** 10.1186/1742-4690-6-89

**Published:** 2009-10-13

**Authors:** Isabel Puigdomènech, Marta Massanella, Cecilia Cabrera, Bonaventura Clotet, Julià Blanco

**Affiliations:** 1 Fundació irsiCaixa-HIVACAT, Institut de Recerca en Ciències de la Salut Germans Trias i Pujol (IGTP), Hospital Germans Trias, Universitat Autònoma de Barcelona, Badalona 08916, Barcelona, Catalonia, Spain; 2 Lluita contra la SIDA Foundation, Institut de Recerca en Ciències de la Salut Germans Trias i Pujol, Hospital Universitari Germans Trias i Pujol, Universitat Autònoma de Barcelona, 08916 Badalona, Barcelona, Spain

## Abstract

Although cell-to-cell HIV transmission was defined in early 90's, in the last five years, several groups have underscored the relevance of this mode of HIV spread between productively infected and uninfected CD4 T cells by defining the term virological synapse (VS). However, unraveling the molecular mechanisms of this efficient mode of viral spread appears to be more controversial than expected. Different authors have highlighted the role of a classical co-receptor-dependent HIV transmission while others describe a co-receptor-independent mechanism as predominant in VS. By analyzing different cellular models (primary cells and cell lines), we suggest that primary cells are highly sensitive to the physical passage of viral particles across the synapses, a co-receptor-independent phenomenon that we call "HIV transfer". Once viral particles are transferred, they can infect target cells by a co-receptor-dependent mechanism that fits with the classical meaning of "HIV transmission" and that is much more efficient in cell lines. Differences in the ability of primary CD4 T cells and cell lines to support HIV transfer and transmission explain most of the reported controversial data and should be taken into account when analyzing cell-to-cell HIV spread. Moreover, the terms transfer and transmission may be useful to define the events occurring at the VS. Thus, HIV particles would be transferred across synapses, while HIV infection would be transmitted between cells. Chronologically, HIV transfer is an early event occurring immediately after the VS formation, which precedes but does not inevitably lead to transmission, a late event resulting in infection.

## Commentary

Cell mediated HIV transmission is a highly efficient mechanism of HIV spread [1]. *In vitro*, mobile lymphocytes do not support efficient HIV replication due to their inability to form cellular conjugates [2], while *in vivo*, most of CD4 T cells are multiply infected, a fact hardly explained by cell-free virus infection [3]. Since the early 90's, cell-to-cell HIV transmission has been intermittently described in the literature [4-6]. However, after the description of the VS formed by HTLV-1 [7], this concept has been widely explored in the study of HIV infection. Initial work defined the structure of HIV-induced VS, in which the viral envelope glycoprotein (gp120/gp41, Env) and its primary receptor CD4, expressed respectively on the surface of infected and uninfected cells, form the central ring [1,8]. Moreover, confocal microscopy showed the crucial role of the cytoskeleton and the recruitment of the viral structural protein Gag and the co-receptor (CXCR4 or CCR5) to the areas of contact between the T cells [8]. More recently, several authors have reported the modulation of the VS by adhesion molecules, although their functional role does not appear to be essential for HIV spread between T cells [9,10]. These results outline the VS as a stable conjugation of infected and uninfected cells involving cell surface receptors. The VS allows for a polarized HIV-release towards the synaptic space wherein viral particles are highly concentrated and efficiently captured by target cells. Besides this, different cellular functions have been explored in the context of cell-to-cell HIV transmission, such as nanotubes or filopodia that may complement the VS, allowing HIV transfer between non-conjugated cells [11,12]. Alternatively, the formation of nanotubes or filopodia into the VS may allow for the exchange of membrane patches between conjugated cells, a phenomenon known as trogocytosis [13], which may play a role in HIV transmission [14].

It has been widely observed that the formation of the synaptic structure is dependent on Env binding to CD4 [9,10,15]. However, the role of the co-receptor during the early events of the VS is still under discussion [16]. A reduction in the function of the VS after co-receptor blockade has been reported [8], suggesting that co-receptor binding precedes Gag co-localization at the VS. However, it has been reported that primary CD4 T cells capture HIV particles by a co-receptor-independent endocytic mechanism [10,15,17,18], and that co-receptor antagonists, fusion inhibitors or neutralizing antibodies directed against the gp41 subunit, do not reduce the amount of HIV particles that cross the VS in several cell-to-cell HIV transmission models [14,15,18]. Again, this is not an unanimous opinion, since other authors do not describe co-receptor-independent capture of HIV [8] or suggest that it is marginal during cell-to-cell HIV transmission [19].

We believe that part of this discrepancy may be explained by the use of primary cells or cell lines as target cells which behave differently in their ability to capture HIV particles and to become productively infected after engaging the VS. To better describe the mechanisms of cell-to-cell HIV transmission in the different target cells, we suggest the use of the term "transfer" when referring to the physical passage of HIV particles from infected to uninfected cells, and we suggest keeping the term "transmission" when this transfer leads to infection of the target cells. Understanding the chronological and causal relationships between HIV transfer and transmission will be required to definitively find out how HIV spreads *in vivo*.

### HIV transfer versus HIV transmission

To date, cell-to-cell HIV transmission to primary CD4 T cells [8,10,14,15,18] and cell lines [15,19] has yielded somewhat discordant results. To understand these differences, we have analyzed two different co-culture models, in which MOLT cells chronically infected with the X4 and R5 isolatesNL4-3 and BaL, were used as effector cells and co-cultured, on the one hand, with primary CD4 T cells or, on the other hand, with the CD4+/CXCR4+ but CCR5- MT-4 cell line. In both models, HIV materials associated with target cells can be measured after intracellular labeling of the Gag antigen (p24) (see additional file 1 for details).

After 2 hours of co-culture, p24 staining can be observed in both the MT-4 cell line and primary CD4 T cells co-cultured with both HIV infected MOLT cells. Addition of the fusion inhibitor C34 did not significantly alter the extent of p24 staining of both target cells whereas the blocking anti-CD4 antibody Leu3a abrogated the capture of p24 antigen. As the X4 and the R5 isolates induced similar HIV transfer regardless of the low or absent CCR5 expression in primary cells and MT-4 cells respectively, HIV appears to be initially transferred to target cells in a co-receptor- and fusion-independent manner [18,20] (additional file 2).

A more complex scenario appears at longer incubation times (24 hours). MT-4 cells co-cultured with MOLT NL4-3 infected cells become totally positive for intracellular p24 antigen with a very high intensity that was vastly reduced by the addition of the fusion inhibitor C34, the blocking anti-CD4 antibody Leu3a (additional file 2) or the Reverse Transcriptase inhibitor AZT (data not shown), confirming the existence of a highly efficient process of productive infection acknowledged as HIV transmission. The remaining fusion-independent p24 staining in the presence of C34 was comparable to that observed after 24 hours of co-culture between MT-4 and BaL-infected MOLT cells and may be identified as the co-receptor-independent transfer of HIV materials (additional file 2). In contrast, primary CD4 T cells behave in a different manner in long duration co-cultures. Primary CD4 T cells which intimately contact NL4-3 infected MOLT cells, die by Env-mediated hemifusion and fusion mechanisms [21,22]. The addition of C34 protects CD4 T cells from dying and at the same time notably increases the amount of p24 transferred to primary CD4 T cells. This suggests that in the absence of gp41 mediated cell death and fusion, HIV transfer is the only consequence arising from the VS. Consistently, the co-culture of primary CD4 T cells with BaL-infected MOLT cells show a high level of p24 transfer to target cells that are unaffected by C34, due to the low impact of cell death and fusion (additional file 2). As reported previously, most of the p24 captured by primary cells at short times is internalized [18]. This observation also applies to MT-4 cells, in which 63+/-5% of p24 captured from MOLT BaL cells at 2 hours are resistant to trypsin, while at 24 hours 50+/-4% remain in trypsin resistant compartments (data not shown, [15]) Of note, at 24 hours the amount of p24 accumulated in primary cells by fusion-independent mechanisms was completely sensitive to the anti-CD4 mAb Leu3a, suggesting that primary CD4 T cells show a particular ability to capture HIV particles after engaging in VS formation. Taken together, these data suggest that MT-4 cells (and by extension other CD4 cell lines) appear to be a suitable model to study HIV transmission. Conversely, the high level of p24 transferred to primary CD4 T cells and their mostly quiescent state are factors that hamper the rapid flow cytometric quantification of infection events making these cells an excellent model to explore the mechanisms involved in early events of HIV transmission, namely HIV transfer.

### On the mechanism of HIV transfer to primary CD4 T cells

An alternative approach to characterizing transport phenomena at the VS is the evaluation of the transfer of membrane lipids from MOLT infected cells to target cells. Membrane transfer to single cells may be the consequence of either hemifusion events between cells [22], or the transfer of viral particles that may fuse with, or may be endocytosed, by target cells. NL4-3 infected MOLT cells induce high level of hemifusion and cell death in primary cells. The addition of C34 blocks gp41-mediated cell death but, as observed for p24 transfer, fail to abrogate membrane capture by primary cells. Similarly, membrane transfer to target cells is also observed for BaL infected cells, and remarkably, is not inhibited by the addition of C34. Of note, membrane transfer is tightly subjected to Env binding to CD4 as it is completely inhibited with the anti-CD4 antibody Leu3a (additional file 3). These data suggest that under conditions in which fusion and hemifusion events are completely blocked membranes are still transferred across the VS. On the other hand, the transfer of membranes from NL4-3 and BaL infected MOLT cells to the MT-4 cell line is lower than that observed in primary cells. Notably, only the transfer of membranes from NL4-3 infected MOLT cells to MT-4 cells is partially inhibited by C34, suggesting that low levels of fusion-dependent events are sufficient to induce an efficient infection of cell lines during cell-to-cell HIV transmission (additional file 3).

Although unstimulated primary CD4 T cells do not produce detectable amounts of HIV particles, they support early events of HIV infection that can be measured by quantifying the amount of newly synthesized HIV DNA by real-time PCR [14,21]. The comparison of HIV infection with p24 and membrane transfer suggest that the latter process follows a CD4-dependent but fusion-independent pathway, while proviral DNA synthesis is completely prevented by both CD4 and gp41 blockade, highlighting the differences between HIV transfer and transmission (additional file 4).

**Figure 1:**
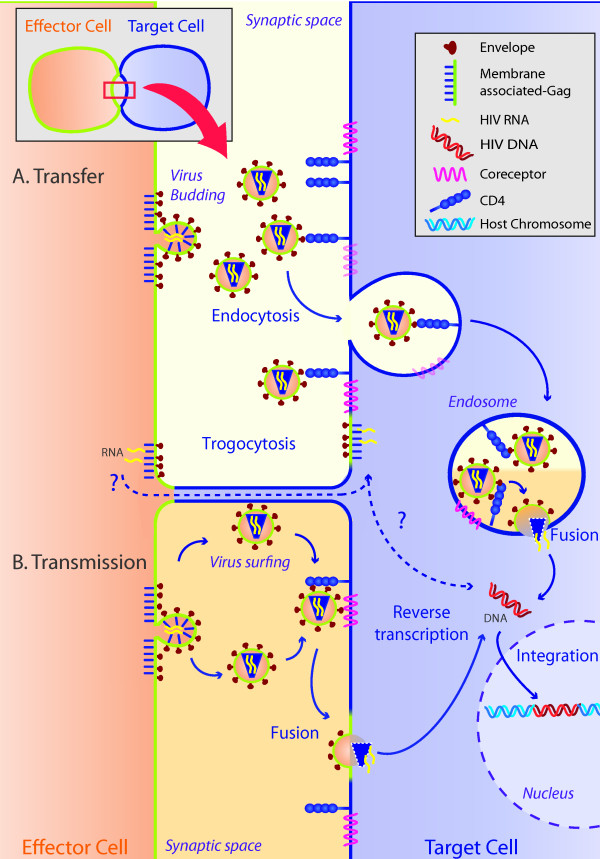
**Steps of HIV transmission**. A portion of the VS (top left) has been enlarged to illustrate HIV transfer (A) and transmission (B). Two mechanisms would be involved in the transfer of HIV materials from infected to target cells after the VS formation. Both are dependent on Env binding to CD4 but independent of co-receptor engagement. First, a massive budding of viral particles from the infected cell (left) to the synaptic space (middle) and a further virion wrapping in the endosomal vesicles by the target cells (right). Second, membrane patches from infected cells carrying HIV budding machinery could be transferred to uninfected cells by trogocytosis through the formation of tethering tubes, potentially allowing for viral RNA to enter the cytoplasm of target cell (without exposure to the extracellular milieu). Furthermore, membrane tubes may help virions to surf extracellularly towards the uninfected cell. For cell-to-cell transmission events (involving infection of target cells), viral particles require both CD4 and the co-receptor, CXCR4 or CCR5, to fuse with the target cell. This process may occur at the plasma membrane or in endosomal compartments, allowing for HIV RNA release into the cytoplasm and initiation of the infectious cycle, after reverse transcription and nuclear import. In the absence of the co-receptor, transferred HIV particles accumulate in the endosomal compartments.

Two non-exclusive mechanisms may explain the fusion-independent transfer at the VS: first, a massive budding of HIV particles that drag membrane components, followed by a massive binding and endocytic capture by the uninfected target cell; and/or second, a trogocytic process involving the transfer of membrane patches carrying HIV budding machinery, a process that is probably linked to the formation of bridged structures (Figure 1). Cumulative evidence suggests that HIV transfer to primary cells follows the first mechanism either by surfing along the membrane bridges or as free particles released into the synaptic space. Electron microscopy data, from an early description [6] to a recent report by Chen *et al*. [20], describe polarized Gag or Gag-GFP budding at the VS and endosomal vesicles containing HIV particles in target cells. Furthermore, biochemical data show that transferred HIV materials are directed towards trypsin resistant compartments [18], and videomicroscopy shows the vesicular transfer of large amounts of Gag, sporadically involving entire synaptic buttons [20]. Although this latter observation may be consistent with trogocytic events, we have described that trogocytosis occurs at the VS from the target to the infected cells, but not in the direction of viral transmission [14]. Of note, trogocytosis, which requires the formation of bridged membrane structures, is observed at the VS before co-receptor engagement, potentially allowing for an open access of the HIV machinery to the cytoplasm of the target cells (Figure 1). However, the complete blockade of infection in the presence of fusion inhibitors rules out the existence of gp41-independent HIV transmission at the VS (additional file 4, [14]).

The differences observed between primary CD4 T cells and a model cell line might be associated with the kinetics of hemifusion/fusion events [23]. Delayed fusion at the cell membrane may favor the duration of hemifusion, increasing endocytosis and cell death events in primary cells. In contrast, rapid fusion kinetics at the cell membrane may favor transmission of HIV infection with lower levels of endocytosis and gp41-mediated death. Assuming that the endocytosis of HIV particles is the main mechanism of HIV transfer in primary CD4 T cells and according to the recent report by Miyauchi *et al*. [24] that describes efficient HIV fusion with endosomal membranes in HeLa cells, a hypothetical sequence of HIV transmission is illustrated in Figure 1. In the absence of functional co-receptor, transfer but not infection occurs; target cells accumulate high amounts of HIV particles in endosomal compartments that fail to induce detectable proviral DNA synthesis. In this case, HIV transfer may be defined as a byproduct of HIV transmission, allowing transferred virions to be released and transmitted to third party cells [18], or alternatively to be processed for antigen presentation. Conversely, in co-receptor expressing target cells, transferred viruses may fuse with the endosomal membranes to reach the cytoplasm and infect the target cells. Some experimental evidence support this endosomal pathway also in primary cells [25]. Irrespective of the entry pathway preferred by HIV during cell-to-cell spread, fusion inhibitors and neutralizing antibodies efficiently block the infectious process [14].

## Conclusion

In summary, evaluation of cell-to-cell HIV transmission is highly dependent on the target cell type employed. Cell lines are a good model to study HIV transmission as they present an activated phenotype that enhances productive infection; however, they show low ability to accumulate HIV particles (HIV transfer) and may not completely mimic primary cells. Freshly isolated primary CD4 T cells are remarkably sensitive to HIV transfer but show low, if any, capability to become productively infected. Given the relevance of primary cells, this limitation can be overcome by monitoring early events of the HIV life cycle such as the synthesis of proviral DNA [14], or the access of HIV cores to the cytoplasm using fluorescent techniques [26]. These differences should be taken into account when analyzing the inhibition of HIV transmission. In particular, the misinterpretation of HIV transfer as HIV transmission should be avoided.

## Competing interests

The authors declare that they have no competing interests.

## Authors' contributions

IP and MM defined the experimental models and performed most of experimental work on HIV transfer; CC contributed to the quantification of proviral DNA; IP, BC and JB designed experiments and wrote the manuscript. All authors read and approved the final manuscript.

## Supplementary Material

Additional file 1 Detailed experimental procedures Click here for file

Additional file 2 HIV transmission and HIV transfer at the VS Click here for file

Additional file 3 Membrane transfer at the VS and association with cell death in primary cells Click here for file

Additional file 4 Quantitative analysis of HIV transfer and transmission to primary CD4 T cells Click here for file

## References

[B1] Piguet V, Sattentau Q (2004). Dangerous liaisons at the virological synapse. J Clin Invest.

[B2] Sourisseau M, Sol-Foulon N, Porrot F, Blanchet F, Schwartz O (2007). Inefficient human immunodeficiency virus replication in mobile lymphocytes. J Virol.

[B3] Dixit NM, Perelson AS (2004). Multiplicity of human immunodeficiency virus infections in lymphoid tissue. J Virol.

[B4] Sato H, Orenstein J, Dimitrov D, Martin M (1992). Cell-to-cell spread of HIV-1 occurs within minutes and may not involve the participation of virus particles. Virology.

[B5] Dimitrov DS, Willey RL, Sato H, Chang LJ, Blumenthal R, Martin MA (1993). Quantitation of human immunodeficiency virus type 1 infection kinetics. J Virol.

[B6] Fais S, Capobianchi MR, Abbate I, Castilletti C, Gentile M, Cordiali Fei P, Ameglio F, Dianzani F (1995). Unidirectional budding of HIV-1 at the site of cell-to-cell contact is associated with co-polarization of intercellular adhesion molecules and HIV-1 viral matrix protein. AIDS.

[B7] Igakura T, Stinchcombe JC, Goon PK, Taylor GP, Weber JN, Griffiths GM, Tanaka Y, Osame M, Bangham CR (2003). Spread of HTLV-I Between Lymphocytes by Virus-Induced Polarization of the Cytoskeleton. Science.

[B8] Jolly C, Kashefi K, Hollinshead M, Sattentau QJ (2004). HIV-1 cell to cell transfer across an Env-induced, actin-dependent synapse. J Exp Med.

[B9] Jolly C, Mitar I, Sattentau QJ (2007). Adhesion molecule interactions facilitate human immunodeficiency virus type 1-induced virological synapse formation between T cells. J Virol.

[B10] Puigdomenech I, Massanella M, Izquierdo-Useros N, Ruiz-Hernandez R, Curriu M, Bofill M, Martinez-Picado J, Juan M, Clotet B, Blanco J (2008). HIV transfer between CD4 T cells does not require LFA-1 binding to ICAM-1 and is governed by the interaction of HIV envelope glycoprotein with CD4. Retrovirology.

[B11] Sherer NM, Lehmann MJ, Jimenez-Soto LF, Horensavitz C, Pypaert M, Mothes W (2007). Retroviruses can establish filopodial bridges for efficient cell-to-cell transmission. Nat Cell Biol.

[B12] Sowinski S, Jolly C, Berninghausen O, Purbhoo MA, Chauveau A, Kohler K, Oddos S, Eissmann P, Brodsky FM, Hopkins C (2008). Membrane nanotubes physically connect T cells over long distances presenting a novel route for HIV-1 transmission. Nat Cell Biol.

[B13] Joly E, Hudrisier D (2003). What is trogocytosis and what is its purpose?. Nat Immunol.

[B14] Massanella M, Puigdomenech I, Cabrera C, Fernandez-Figueras MT, Aucher A, Gaibelet G, Hudrisier D, Garcia E, Bofill M, Clotet B, Blanco J (2009). Antigp41 antibodies fail to block early events of virological synapses but inhibit HIV spread between T cells. AIDS.

[B15] Chen P, Hubner W, Spinelli MA, Chen BK (2007). Predominant mode of human immunodeficiency virus transfer between T cells is mediated by sustained Env-dependent neutralization-resistant virological synapses. J Virol.

[B16] Martin N, Sattentau Q (2009). Cell-to-cell HIV-1 spread and its implications for immune evasion. Curr Opin HIV AIDS.

[B17] Ruggiero E, Bona R, Muratori C, Federico M (2008). Virological consequences of early events following cell-cell contact between human immunodeficiency virus type 1-infected and uninfected CD4+ cells. J Virol.

[B18] Blanco J, Bosch B, Fernandez-Figueras MT, Barretina J, Clotet B, Este JA (2004). High level of coreceptor-independent HIV transfer induced by contacts between primary CD4 T cells. J Biol Chem.

[B19] Sol-Foulon N, Sourisseau M, Porrot F, Thoulouze MI, Trouillet C, Nobile C, Blanchet F, di Bartolo V, Noraz N, Taylor N (2007). ZAP-70 kinase regulates HIV cell-to-cell spread and virological synapse formation. EMBO J.

[B20] Hubner W, McNerney GP, Chen P, Dale BM, Gordon RE, Chuang FY, Li XD, Asmuth DM, Huser T, Chen BK (2009). Quantitative 3D video microscopy of HIV transfer across T cell virological synapses. Science.

[B21] Blanco J, Barretina J, Clotet B, Este JA (2004). R5 HIV gp120-mediated cellular contacts induce the death of single CCR5-expressing CD4 T cells by a gp41-dependent mechanism. J Leukoc Biol.

[B22] Blanco J, Barretina J, Ferri KF, Jacotot E, Gutierrez A, Armand-Ugon M, Cabrera C, Kroemer G, Clotet B, Este JA (2003). Cell-surface-expressed HIV-1 envelope induces the death of CD4 T cells during GP41-mediated hemifusion-like events. Virology.

[B23] Melikyan GB (2008). Common principles and intermediates of viral protein-mediated fusion: the HIV-1 paradigm. Retrovirology.

[B24] Miyauchi K, Kim Y, Latinovic O, Morozov V, Melikyan GB (2009). HIV enters cells via endocytosis and dynamin-dependent fusion with endosomes. Cell.

[B25] Clotet-Codina I, Bosch B, Senserrich J, Fernández-Figueras MT, Peña R, Ballana E, Bofill M, Clotet B, Este JA (2009). HIV endocytosis after dendritic cell to T cell viral transfer leads to productive virus infection. Antiviral Research.

[B26] Cavrois M, De Noronha C, Greene WC (2002). A sensitive and specific enzyme-based assay detecting HIV-1 virion fusion in primary T lymphocytes. Nat Biotechnol.

