# Supplementary material for: On the steps of cell-to-cell HIV transmission between CD4 T cells
Source: Retrovirology. 2009 Oct 13;6:89. doi: 10.1186/1742-4690-6-89 (PMC2768678; doi:10.1186/1742-4690-6-89)
Supplement: Additional file 1 — Detailed experimental procedures [file 1742-4690-6-89-S1.PDF]

Detailed experimental procedures.

MOLT cells uninfected or chronically infected with recombinant HIV isolates bearing the NL4-3 (X4) or the BaL (R5) envelopes have been previously described [1]. Infected cells are 90 % p24+ and express similar levels of HIV envelope. Primary CD4 T cells were isolated by immunomagnetic negative selection (Miltenyi Biotec) of PBMCs obtained from healthy donors through the local blood bank. Final preparations were >95% of CD4 positive T cells as assessed by flow cytometry. Primary CD4 T cells were used without any further treatment in coculture experiments. MT-4 cells were obtained from Dr. D Richman through the AIDS Research and Reference Reagent Program. Before coculturing with MOLT cells, MT-4 cells were stained with 10 µg/µL of the far red cell tracer DDAO (Molecular Probes/Invitrogen) for 30 min at 37ºC in the dark. For membrane transfer experiments, MOLT cells were stained with 34 µM of the green lipophylic probe DiO (Molecular Probes/Invitrogen) for 15 min at 37ºC. Cells were extensively washed before use in order to reduce non specific DiO transfer.

Cocultures of infected and target cells (ratio 1:1) were performed in 96 well plates by mixing 200.000 cells of each type in a final volume of 200 µL. The fusion inhibitor C34 (5 µg/mL, AIDS Research and Reference Reagent Program) and the anti CD4 mAb Leu3a (0,25 µg/mL, BD Biosciences) were used as inhibition controls. Cocultures of target cells with uninfected MOLT cells were performed in each experiment. Membrane (DiO) and gag (p24) transfer were analyzed by flow cytometry. For DiO transfer cells were fixed in Formaldehyde 1% and acquired in a FACScalibur flow cytometer. For p24 staining, cells were fixed and then simultaneously permeabilized (Fix and Perm reagent, Invitrogen) and stained with the PE labeled anti-HIV p24 antibody Kc57 (Coulter). For trypsin treatment, cells were washed with PBS-EDTA solution (Versene, Invitrogen) and treated for 10 min at RT with 0.25 % trypsin solution (Invitrogen). Trypsin action was stopped by addition of fetal calf serum. Cells were then washed with PBS and stained as indicated above. Trypsin treatment removes >90% of cell-surface CD4 staining [2]. Quantification of HIV transfer was assessed by the ratio of Mean Fluorescence Intensity (MFI) of infected and uninfected samples.

To analyze transfer to target cells, living primary CD4 T cells have been gated separately from MOLT-4 cells according to morphological parameters. Living MT-4 cells, which are morphologically similar to MOLT cells, were identified by their DDAO staining. Conjugates or syncytia formed between MT-4 and MOLT-4 cells were excluded from the analysis as they present a larger size and the combined fluorescence of both cells (i.e DDAO and p24 positive).

The synthesis of proviral DNA in cocultures of MOLT cells with primary CD4 T cells was assessed by qPCR as described [3]. Primary CD4 T cells (500,000 cells) were cultured in 48-well plate with 500,000 infected MOLT cells. After 24 h, DNA was extracted using the Qiamp DNA Blood mini kit (Qiagen) and amplified in triplicate using a Taqman universal PCR Master Mix, primers and probes for HIV and CCR5 (Applied Biosystems). Reactions were conducted in an ABI 7000 Sequence Detection System (Applied Biosystems). Relative proviral DNA synthesis was calculated using the $2^{-\Delta\Delta CT}$ method [4]. $\Delta\Delta CT = (CT_{HIV}-CT_{CCR5})_{Test}-(CT_{HIV}-CT_{CCR5})_{Control}$, where CT is the fractional cycle number that reaches a fixed threshold.

References

1- Blanco J, Barretina J, Clotet B, Este JA: **R5 HIV gp120-mediated cellular contacts induce the death of single CCR5-expressing CD4 T cells by a gp41-dependent mechanism.** *J Leukoc Biol* 2004, **76:**804-811.

2- Blanco J, Bosch B, Fernandez-Figueras MT, Barretina J, Clotet B, Este JA: **High level of coreceptor-independent HIV transfer induced by contacts between primary CD4 T cells.** *J Biol Chem* 2004, **279:**51305-51314.

3- Massanella M, Puigdomenech I, Cabrera C, Fernandez-Figueras MT, Aucher A, Gaibelet G, Hudrisier D, Garcia E, Bofill M, Clotet B, Blanco J: **Antigp41 antibodies fail to block early events of virological synapses but inhibit HIV spread between T cells.** *AIDS* 2009, **23:**183-188.

4- Livak KJ, Schmittgen TD. **Analysis of relative gene expression data using real-time quantitative PCR and the 2(-Delta Delta C(T)) Method**. Methods 2001,25:402-408.
