# Supplementary material for: On the steps of cell-to-cell HIV transmission between CD4 T cells
Source: Retrovirology. 2009 Oct 13;6:89. doi: 10.1186/1742-4690-6-89 (PMC2768678; doi:10.1186/1742-4690-6-89)
Supplement: Additional file 2 — HIV transmission and HIV transfer at the VS [file 1742-4690-6-89-S2.PDF]

Additional file 2

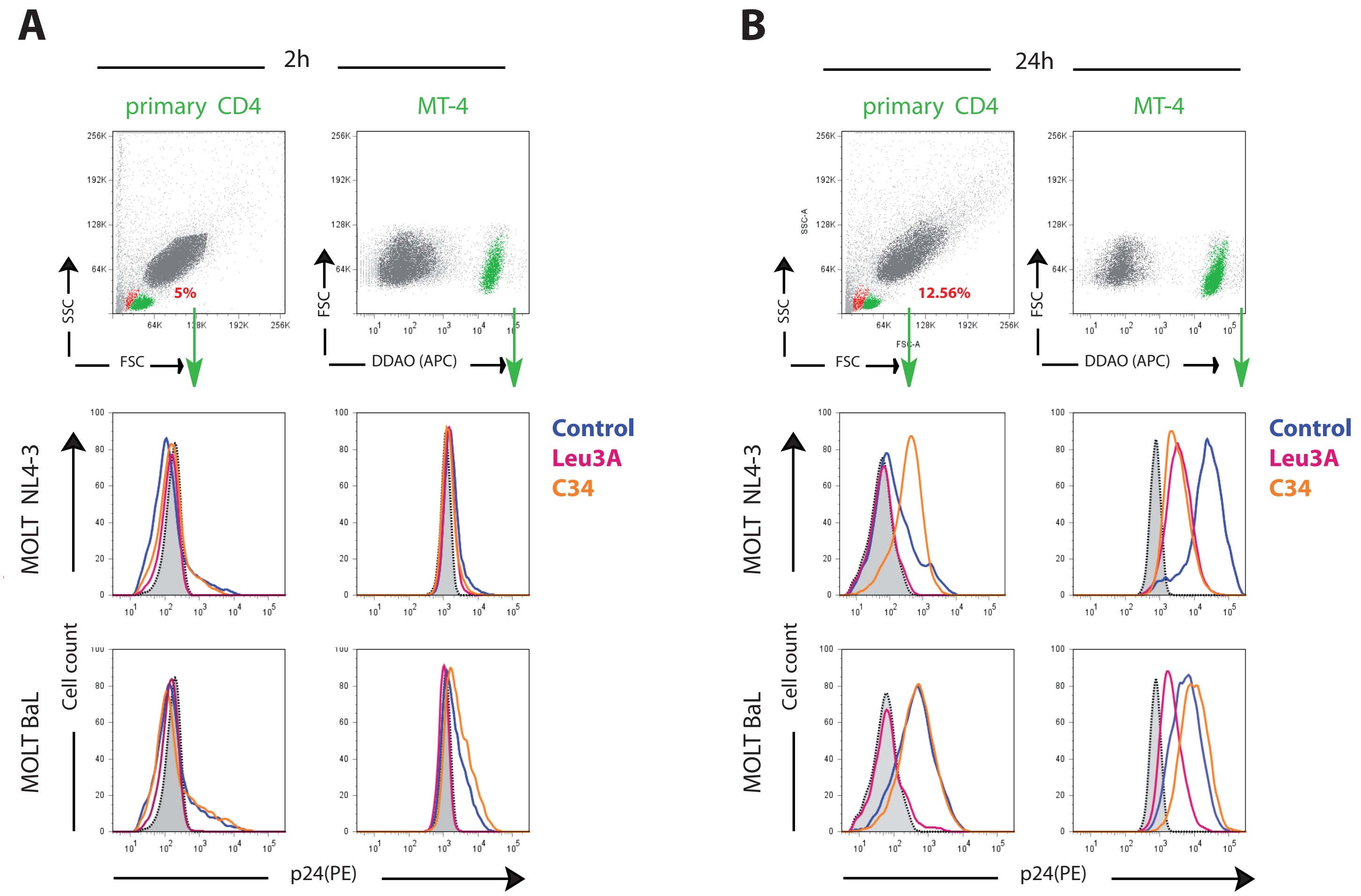

Additional file 2. HIV transmission and HIV transfer at the VS. MOLT cells uninfected or chronically infected with NL4-3 or BaL isolates were cocultured either with the DDAO labeled MT-4 cells or with purified primary CD4 T cells. Target cells were identified by DDAO staining or morphological parameters. In each panel, upper plots show the morphology of MOLT-NL4-3 cells cocultured with target cells (green populations). Values representing the % of dead target cells (red populations) are shown for primary cells. The level of p24 antigen was measured by intracellular staining using the KC57 mAb after 2-hours (A) or 24-hours (B) of coculture. Histograms show p24 staining in gated living target cells in the absence (blue) or the presence of the fusion-blocking peptide C34 (orange) and the anti-CD4 antibody Leu3A (dark red). Grey tinted peaks show the staining of uninfected control cocultures. A single representative experiment is shown
