# Supplementary material for: On the steps of cell-to-cell HIV transmission between CD4 T cells
Source: Retrovirology. 2009 Oct 13;6:89. doi: 10.1186/1742-4690-6-89 (PMC2768678; doi:10.1186/1742-4690-6-89)
Supplement: Additional file 3 — Membrane transfer at the VS and association with cell death in primary cells [file 1742-4690-6-89-S3.PDF]

Additional file 3

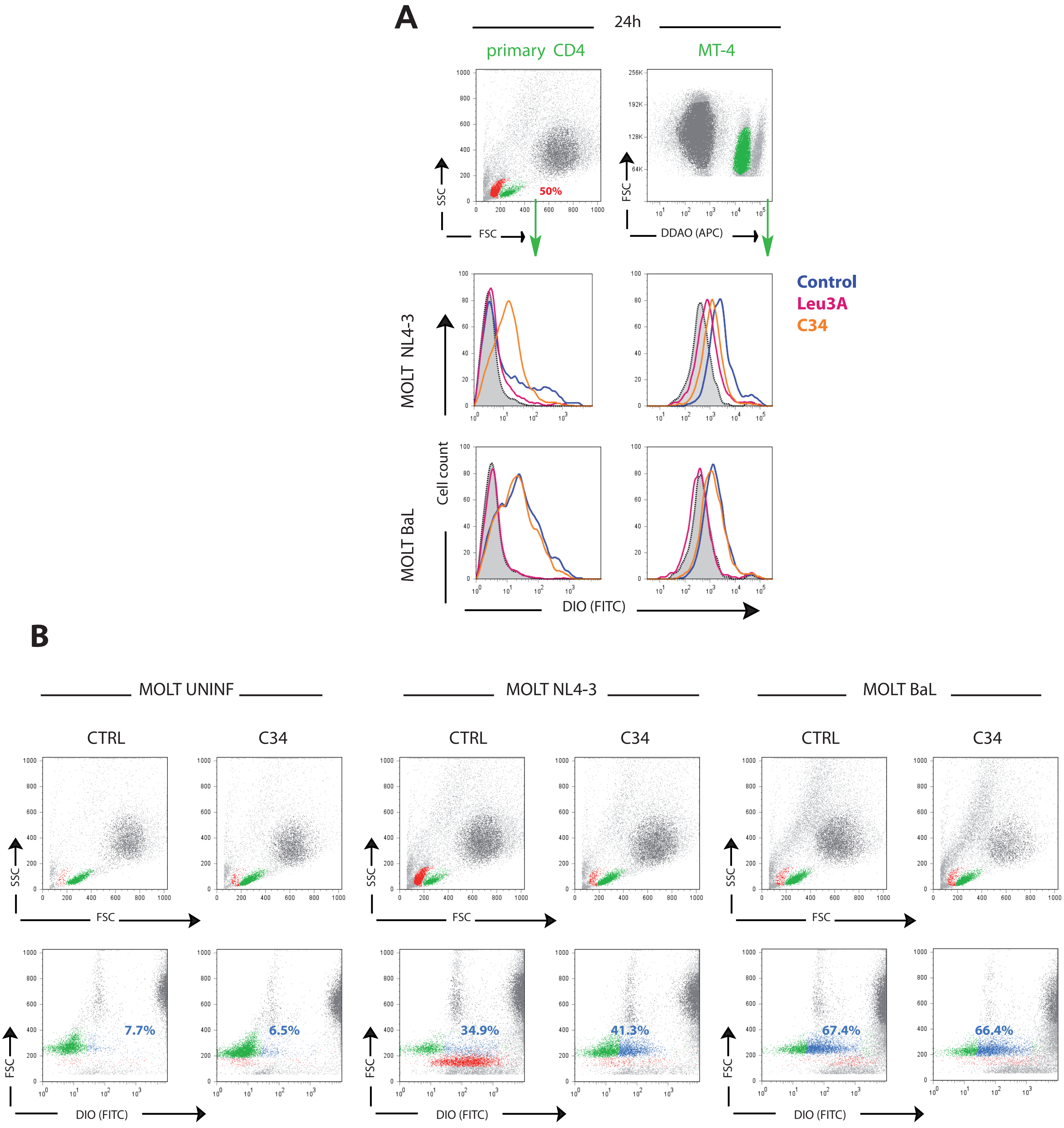

Additional file 3. Membrane transfer at the VS and association with cell death in primary cells. (A) MOLT cells uninfected or chronically infected with NL4-3 or BaL isolates were labeled with the membrane probe DIO before coculture either with the DDAO labeled MT-4 cells or with purified primary CD4 T cells. At 24-hours, membrane transfer to living target cells (gated in green in upper plots) was analyzed. Histograms illustrate the transfer from MOLT NL4-3 or MOLT BaL cells in the absence (blue) or the presence of the fusion peptide C34 (orange) and the anti-CD4 antibody Leu3A (dark red). Grey tinted peaks show the staining of uninfected control cocultures. A single representative experiment is shown. (B) Upper dot-plots illustrate the morphology of primary CD4 T cells cocultured with the indicated MOLT cells for 24-hours. Living CD4 T cells appear in green and dead CD4 T cells in red. Forward scatter and DiO staining plots, corresponding to the same cocultures are represented below to illustrate the cellular distribution of the fluorescent probe. Note that in the coculture of CD4 T cells with NL4-3 infected MOLT cells, dead CD4 T cells show the highest levels of DiO transfer (red). The addition of C34 blocks hemifusion events enhancing fusion-independent membrane transfer. Values correspond to the percentage of DiO positive cells (in blue) in the living CD4 T cell gate.
