# Supplementary material for: On the steps of cell-to-cell HIV transmission between CD4 T cells
Source: Retrovirology. 2009 Oct 13;6:89. doi: 10.1186/1742-4690-6-89 (PMC2768678; doi:10.1186/1742-4690-6-89)
Supplement: Additional file 4 — Quantitative analysis of HIV transfer and transmission to primary CD4 T cells [file 1742-4690-6-89-S4.PDF]

Additional file 4

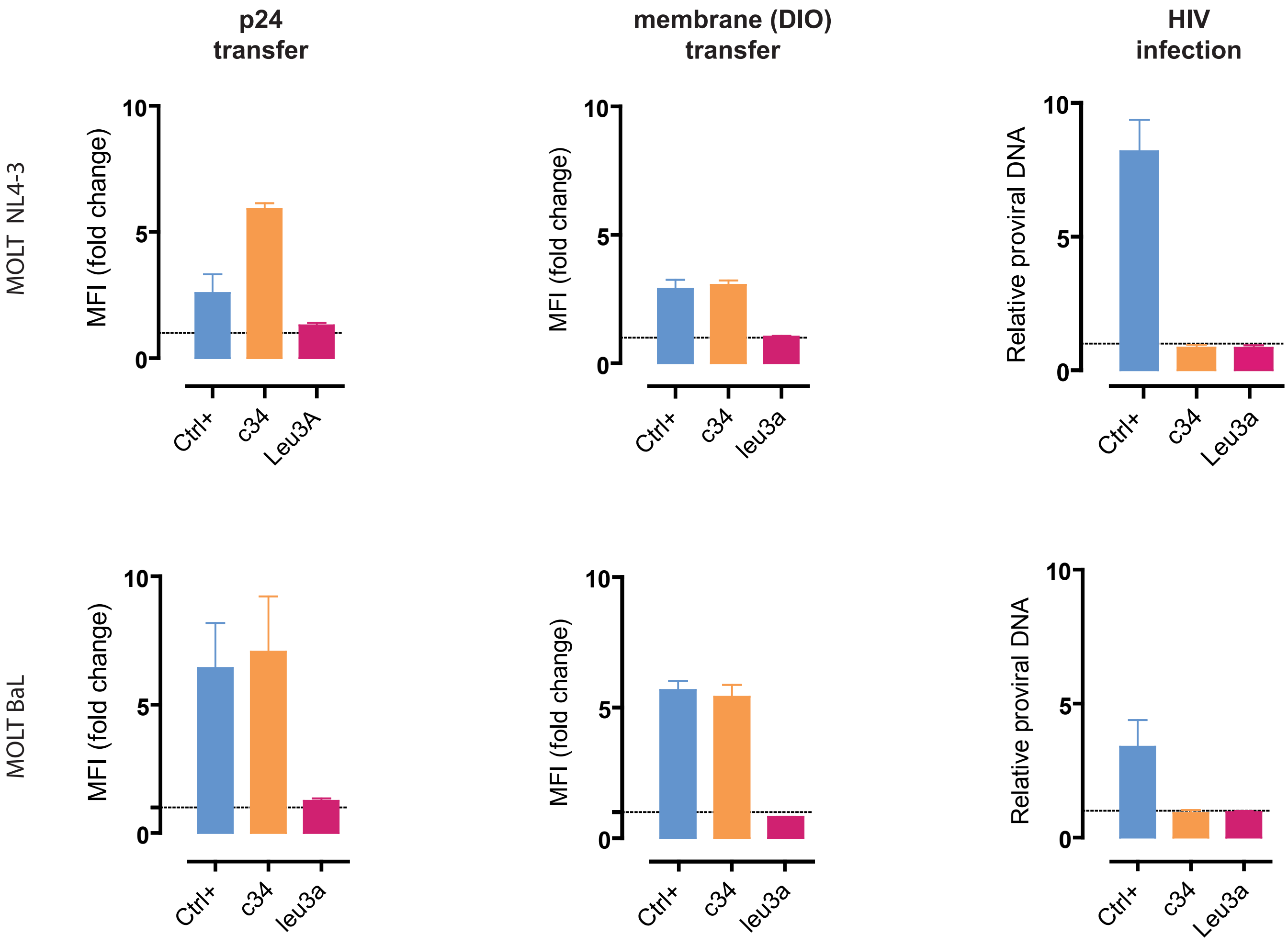

Additional file 4. Quantitative analysis of HIV transfer and transmission to primary CD4 T cells. Transfer of p24 antigen (left), and membrane (middle) and DNA synthesis (right) after 24-hours of culture between MOLT NL4-3 cells (top) or MOLT BaL cells (bottom) and primary CD4 T cells in the absence of inhibitors (blue bars) and the presence of C34 (orange bars) or the anti-CD4 antibody Leu3a (purple bars). Data are Mean+/-SD of three different experiments and show the fold change from uninfected cocultures in fluorescence intensity of the living target cells (for p24 and DiO transfer) or the fold change from a culture of MOLT cells alone to measure the relative proviral DNA quantification (for HIV infection).
